# Plasma miR‐21 as a potential predictor in prediabetic individuals with a positive family history of type 2 diabetes mellitus

**DOI:** 10.14814/phy2.15163

**Published:** 2022-01-25

**Authors:** Zakieh Yazdanpanah, Nasrin Kazemipour, Seyed Mehdi Kalantar, Mohammad Yahya Vahidi Mehrjardi

**Affiliations:** ^1^ Biochemistry Division Department of Basic Science School of Veterinary Medicine, Shiraz University Shiraz Iran; ^2^ Department of Medical Genetic Medical School Shahid Sadoughi University of Medical Science Yazd Iran; ^3^ Medical Genetics Research Center Shahid Sadoughi University of Medical Science Yazd Iran

**Keywords:** family history of type 2 diabetes mellitus, microRNA‐126‐3p, microRNA‐21, prediabetes

## Abstract

Type 2 diabetes mellitus (T2DM) is a heritable metabolic perturbation, rapidly growing across the world. Primary recognition of susceptible individuals with a family history of type 2 diabetes (FHD) in the prediabetes stage could delay the onset of T2DM or reduce complications induced by diabetes. This study aims to evaluate the expression levels of miR‐21, miR‐126 as noninvasive predictive biomarkers in individuals with genetic predisposition and investigate the correlation of miRNAs and cardiometabolic risk factors. Our study demonstrated that miR‐21 expression has a notable elevate in both groups of T2DM and pre‐T2DM. miR‐21 expression was distinguished in the pre‐T2DM and T2DM from the nondiabetic individuals by ROC curve analysis with AUC of 0.77 (95% CI 0.65–0.90; *p *= 0.0004) and AUC of 0.78 (95% CI 0.64–0.92; *p *= 0.0042), respectively. The relative gene expression of miR‐126 was nearly equal among groups. miR‐21 expression was positively associated with glycosylated hemoglobin (HbA1c), fasting blood sugar (FBS), and triglyceride (TG) and might have diagnostic value for T2DM and pre‐T2DM. This study has revealed that the expression level of miR‐21 can be considered as a non‐invasive and rapid tool for distinguishing pre‐T2DM and T2DM counterparts from healthy individuals.

## Graphical abstract

MicroRNAs in pre‐T2DM and T2DM: miR‐21 as a potential biomarker in pre‐T2DM and T2DM.

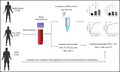

**Main Points**

- The relative expression of miR‐21 and miR‐126 was assessed in individuals with genetic susceptibility to T2DM.
- The expression level of miR‐21 was upregulated in pre‐T2DM and T2DM groups. The expression of miR‐126 was not altered among groups. The approximately equal diagnostic value in T2DM and pre‐T2DM groups could present miR‐21 as a potential ideal biomarker in the prediabetic phase.
- The positive correlation between miR‐21 and HbA1c, FBS, and TG could be an early predictor of disease onset.

## 1 INTRODUCTION

The currently increasing prevalence of T2DM is a considerable global concern in healthcare that will rise to approximately 642 million by 2040 (Al‐Lawati, 2017).T2DM is a chronic metabolic syndrome, characterized by impaired either insulin secretion or resistance to actions of insulin, or both, resulting in hyperglycemia (Stumvoll et al., 2005). T2DM is a multifactorial disorder in which numerous genetic variations and environmental factors are involved in its occurrence and pathogenesis (Mambiya et al., 2019; Schellenberg et al., 2013). Genetic predisposition plays an important role in the development of T2DM. The character of the intricate T2DM polygenic has been presented in several genome‐wide association studies (GWAS). Individuals with a genetic positive history are more susceptible to T2DM (38%–70%) (Fuchsberger et al., 2016; McCarthy, 2010).

Microvascular and macrovascular complications mediated by T2DM, including retinopathy, neuropathy, nephropathy, and atherosclerosis, gradually initiate and progress during the prediabetes phase (Association AD, 2016). Prediabetes is a complication with no clinical manifestations and mediocre hyperglycemia condition that generally occurs because of impaired glucose tolerance. Around 25% of people with prediabetes will develop T2DM within 3–5 years. There is a hypothesis that genetic predisposition has a notable effect on the progression of prediabetes to diabetes (Hostalek, 2019; Sidorkiewicz et al., 2020). Therefore, it is necessary to identify susceptible individuals within the prediabetes phase.

In recent years, microRNAs (miRNAs) are attention as ideal molecular biomarkers in various research. miRs are a highly conserved of small non‐coding RNAs, that by protein translation repression or mRNA destabilization regulate gene expression in post‐transcriptional (Sadik et al., 2018). Many studies have demonstrated that miRs are expressed in various tissues and cell types and present a constant and tissue‐specific expression pattern (Bartel, 2009). Moreover, miRs involved in regulating several important cellular functions, including cell cycle regulation, differentiation, apoptosis, and maintenance of the immune system cells repertoire (Bartel, 2004). Alteration in miRs should be considered to the diagnosis of diseases like various cancer (Leão et al., 2021; Tanaka et al., 2009; Zheng & Hou, 2021) and cardiovascular diseases (Fichtlscherer et al., 2010). An increasing number of miRNAs profiles are correlated with the development and pathology of type 2 diabetes mellitus (T2DM) and the homeostasis of glucose and lipid metabolism (Hashimoto & Tanaka, 2017; Lagos‐Quintana et al., 2001; Sebastiani et al., 2017). Dysregulation of miRNAs commences years before imbalanced blood glucose, as an example, miR‐491‐5p, miR‐1307‐3p, and miR‐298 were identified years before the onset of diabetes, those biomarkers may be the appropriate noninvasive tools for prediabetic and diabetic patients (Sidorkiewicz et al., 2020). Liu et al. (2014) and Yang et al. (2014) suggested that miR‐21 and miR‐126 were strongly associated with T2DM. miR‐21 or hsa‐miR‐21 is recurrently overexpressed in multiple diseases, suggesting that it harbors a key role in cell proliferation and apoptosis (Krichevsky & Gabriely, 2009). It is been reported that miR‐21 controls adipogenic differentiation and proliferation by modulation of the TGF‐β pathway and phosphatase and tensin homolog deleted on chromosome ten (PTEN). PTEN plays an important role in the downregulation of insulin signaling by PI3K pathway in 3T3‐L1 adipocytes (Nakashima et al., 2000; Roy et al., 2009; Tang et al., 2005). Moreover, several studies have demonstrated an association between miR‐21 and diabetic complications such as retinopathy and nephropathy. miR‐126 expression is abundant in endothelial cells which maintain endothelial homeostasis and restores vascular integrity and angiogenesis, and its down‐expression leads to T2DM‐mediated cardiovascular diseases by targeting gene SPRED1 (Fish et al., 2008; Meng et al., 2012). The aim of this study was to investigate the expression levels of miR‐126 and miR‐21 in plasma samples of healthy subjects, and pre‐T2DM and T2DM patients also assessed these molecules as noninvasive diagnostic tools for the identification of susceptible individuals with diabetes.

## 2 MATERIALS AND METHODS

### 2.1 Study population

Eighty‐two individuals aged 45–70 years were recruited from the Diabetes Research Centre, Yazd, Iran, who were divided into control (FBS: 4.8–5.2 mmol/l; HbA1c, <5.7%), pre‐T2DM (FBS, 6.1–6.9 mmol/l; HbA1c 5.7%–6.4%), and T2DM (FBS ≥7.0 mmol/l; HbA1c ≥ 6.5%) groups. The diagnostic criteria for T2DM and pre‐T2DM were detected supported the 2020 American Diabetes Association Standards of Medical Care (Association AD, 2020). It is a reality that several factors, including psychological stress, nutrition (dietary pattern), physical activity, inflammatory factors, etc., affect the expression of miRs, thus we tried to minimize the environmental factors influencing the expression of microRNAs and the exclusion criteria for study participants included: being under physical or medical treatment, a history of liver cirrhosis or malignancy, a body mass index (BMI) of upper than 40 kg/m^2^, having complications of DM, including nephropathy, retinopathy, neuropathy, and cardiovascular disorders, having chronic kidney, liver, lung, and chronic or acute inflammatory diseases (especially acute inflammation of the pancreas and endocarditis), heart valve disease, short bowel syndrome (SBS) and allergies, pregnancy or breastfeeding women, suffering from kidney, liver, heart disease, and any autoimmune disorders that will exert a hidden effect on the miRs level.

### 2.2 Data collection and measurements

T2DM patients were obtained from the presence of known family members with type 2 diabetes mellitus in first‐degree relatives (siblings, parents, or grandparents). The disease history, age, gender, systolic blood pressure (SBP), diastolic blood pressure (DBP), and glycemic control were recorded.

Fasting blood sugar (FBS) levels were measured by routine enzymatic methods. Glycated hemoglobin (HbA1c) was assessed colorimetrically (Biosystems, Barcelona, Spain). Total cholesterol (TC) was measured by an enzymatic colorimetric test (GPO‐PAP method); high‐density lipoprotein cholesterol (HDL‐C) levels were detected by a BA 400 analyzer (BioSystems; Spain); and the LDL‐C was calculated using the traditional Friedewald's formula (FF), LDL‐C = (TC) – (HDL‐C) – (TG/5). The clinical parameters of the individuals are indicated in Table 1.

**TABLE 1:** Comparisons of relative expression miRs (miR‐21 and miR‐126), clinical and biochemical characteristics of individuals in each group

Group	Gender (M/F)	Age (year)	Relative Expression miR−21	Relative Expression miR−126	FBS (mmol/l)	HbA1c (%)	TG (mg/dl)	TC (mg/dl)	LDL‐C (mg/dl)	HDL (mg/dl)	SBP (mm Hg)	DBP (mm Hg)
Control	19/10	50.42 ± 6.14	1.13 ± 1.02	1.37 ± 1.53****	5.29 ± 0.58****	5.15 ± 0.54	137.79 ± 23.90	151.55 ± 24.15	72.51 ± 23.58	51.48 ± 7.68	120.17 ± 12.43^a^	78.27± 9.56^a^
Pre‐T2DM	18/11	48.97 ± 9.21	2.18 ± 1.11*	1.93 ± 2.08****	6.25 ± 1.46* ^,^ ***	6.21 ± 0.73* ^,^ ***	177.92 ± 42.30* ^,^ ***	207.03 ± 31.12* ^,^ ***	123.22 ± 36.67*	43.82 ± 6.49*	125.00 ± 11.05^a^	83.39± 11.22^a^
T2DM	15/9	54.42 ± 7.76	2.90 ± 1.64*	1.77 ± 2.38****	9.12 ± 1.19* ^,^ **	7.16 ± 0.16* ^,^ **	220.12 ± 29.28* ^,^ **	231.48 ± 47.91* ^,^ **	143.14 ± 44.92*	42.84 ± 7.95*	131.87 ± 18.5^a^	83.12± 10.71^a^ ^,^ *

Note

The data are presented as mean ±SD and analyzed by one‐way ANOVA (Bonferroni's multiple comparisons test).

Abbreviations: DBP, diastolic blood pressure; F, female; FBS, fasting blood sugar; HbA1c, glycosylated hemoglobin; HDL‐C, high‐density lipoprotein cholesterol; LDL‐C, low‐density lipoprotein cholesterol; M, male; SBP, systolic blood pressure; TC, total cholesterol.

^a^ Nonparametric Kruskal–Wallis.

* Compared with control group, *p* < 0.05

** compared with pre‐T2DM group, *p* < 0.05

*** compared with T2DM group, *p* < 0.05

**** *p*‐value was obtained after variables were log transformed to normalize the distributions.

### 2.3 Quantitative real‐time PCR assay

Total RNA (including miRNAs) was purified from the plasma samples briefly: The samples were instantly centrifuged at 3000 g for 10 min at 4°C. Subsequently, the supernatant phase (the plasma) was carefully removed and stored at −80°C until further analyses. The plasma samples were thawed on ice. Five hundred microliters of each one of the plasma samples was used for RNA extraction. Total RNA was purified from plasma using Trizol LS reagent (Thermo Fisher Scientific, USA), according to the product protocol. The quality and quantity of purified RNA were evaluated by the A260/A280 and 260/230 ratio with a Nanodrop spectrophotometer (Thermo Scientific, Wilmington, USA). MiRNA cDNA synthesis was performed using the BONmiR kit (Stem Cell Technology, Iran), consistent with the manufacturer's manual. In this study, the 2^−ΔΔCt^ method was computed as relative gene expression. SNORD47 was used as an internal control. Primer sequences of miR‐21, miR‐126, and SNORD47 were 5‐ GCCCGCTAGCTTATCAGACTGATG −3 and 5‐GTCCGCTCGTACCGTGAGTAATA‐3 and 5‐ATCACTGTAAAACCGTTCCA‐3, respectively. Reverse primers were provided from the Bonyakhte Company (Stem Cell Technology, Iran). The real‐time PCR reaction was accomplished using the BONmiR QPCR Kit (Stem Cell Technology, Iran) according to the following compounds: 1 μl cDNA, 0.5 μl miRNA‐specific forward primer, 0.5 μl universal reverse primer, 6.5 μl miRNA QPCR master mix, and 4.5 μl nuclease‐free water for every one of the reactions with 13 μl of a terminal volume. RT‐qPCR steps included initial denaturation at 94°C for 5 min, followed by 40 cycles of denaturation at 94°C for 5 s, and annealing and extension at 60°C for 40 s. The melting curve was considered by increasing the temperature from 65 to 95°C to guarantee that no primer dimers or unwanted genomic DNA are produced. All reactions were performed in triplicates on 48‐well plates (Applied Biosystems, Step One Plus, USA). For more accuracy, the efficiency of the primer was calculated by a standard curve which the logarithm of cDNA serial dilutions of a control sample for each miRs (miR‐126 and miR‐21), and SNORD were against Ct values, then primer efficiency determines with the following formula: 10 (−1/slope)‐1. The specificity of primers was assessed using the melting curves.

### 2.4 Statistical analysis

Statistical analysis was calculated using IBM SPSS Statistics, version 19 (IBM Corp, Armonk, NY, USA); data are shown as mean ±SD, and *p* < 0.05 was considered significant. Normally plus non‐normally distributed continuous data were assessed by the Kolmogorov–Smirnov and the Shapiro–Wilk tests. miR‐126 and FBS variables were normalized by logarithmic transformation, and significance was calculated by parametric analysis. Analysis of variance (ANOVA) was conducted to compare relative gene expression/the clinical features (excepting DBP and SBP), followed by the Bonferroni's multiple comparisons test. The correlation between the expression miRs and biochemical parameters was carried out using the Pearson correlation and the Spearman test (DBP and SBP) analysis. Multiple linear regression analysis (two‐sided, α = 0.05) was employed to analyze the principal factors influencing miR‐21 and miR‐126 expressions with relevant indicators as independent variables. Receiver operating characteristic (ROC) curves and the area under the ROC curve (AUC) were used to assess the diagnostic value of miR‐21 in pre‐T2DM and T2DM groups. Considering the ROC analysis, the best statistical cutoff amounts of miRs were measured, and the sensitivity and specificity for particular cutoff points were then attained. Results with *p *< 0.05 were regarded as statistically significant.

## 3 RESULTS

### 3.1 Clinical and laboratory biochemistry variables

The clinical and biochemical parameters of study participants are illustrated in Table 1. A statistically significant difference in HbA1c, FBS, and TG was observed among the three study groups (all *p *< 0.05). LDL‐C, HDL, and TC levels were significantly increased in the T2DM group compared with control group (all *p *< 0.05); however, there was no significant difference between the pre‐T2DM with T2DM and control groups (*p *> 0.05), showing a gradual enhancement trend from the control group to the pre‐T2DM group, and then to the T2DM group. Besides, there was no significant difference in gender, age, SBP, and DBP levels among the T2DM, pre‐T2DM, and control groups (all *p *> 0.05).

### 3.2 The efficiency of primers

The real‐time PCR assay demonstrated the linearity between the logarithmic values of the miRNAs and the Ct values. The efficiency of miR‐21, miR‐126, and SNORD was calculated as 1.9 (R^2^ = 0.98), 1.94 (R^2^ = 0.98), and 2.04 (R^2^ = 0.99), respectively.

### 3.3 Relative expression of miR‐21 and miR‐126

**FIGURE 1:**
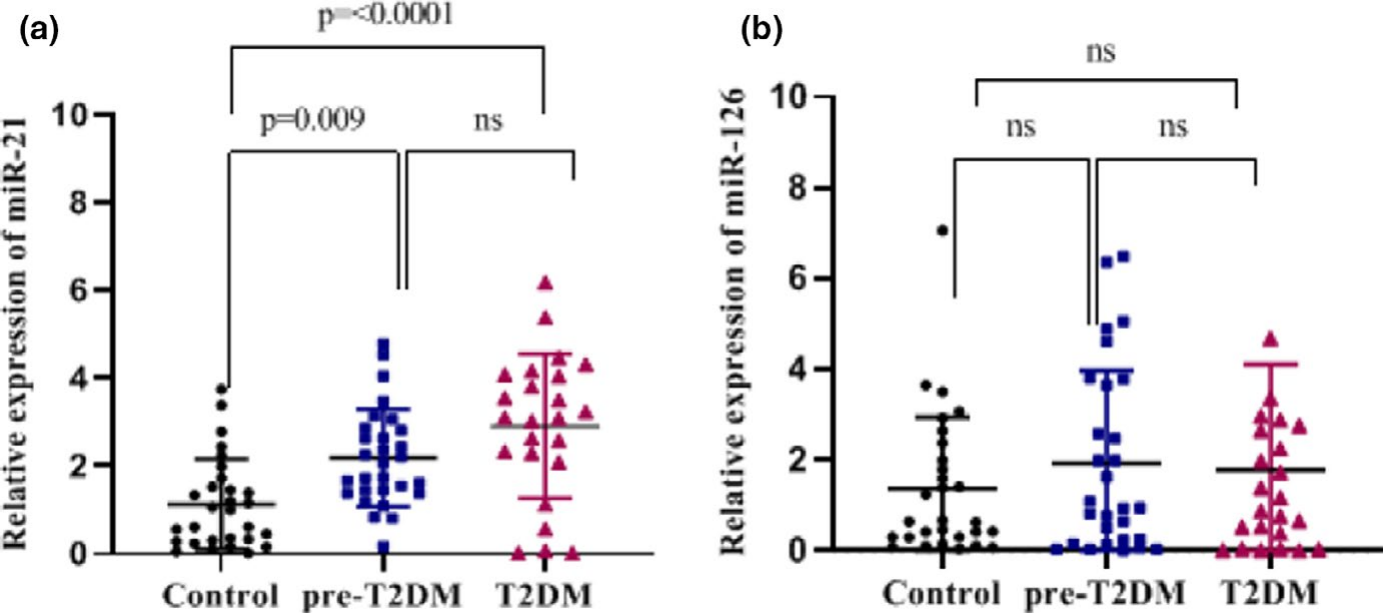
Relative expression of miR‐21 (a) and miR‐126 (b) in the plasma samples of healthy, pre‐T2DH, and T2DH determined by RT‐qPCR. After the data were normalized with SNORD47 (as an internal control), the expression of miR‐21 and miR‐126 was calculated by fold change. The data were presented as mean ±SD and analyzed by one‐way ANOVA (Bonferroni's multiple comparisons test). miR‐21 expression was significantly increased in pre‐T2DM and T2DM patients compared with the healthy group, while no significant difference was observed between pre‐T2DM and T2DM groups. There was no statistically significant difference between the three groups regarding the expression of miR‐126. Bars represent mean. Error bars represent SD

miR‐21 and miR‐126 relative expressions of individuals in each group (x ± S.D.) are demonstrated in Table 1. The data were normalized by SNORD as an internal control and the expression levels for the three groups were calculated using fold change. miR‐21 expression was significantly upregulated in plasma samples of the pre‐T2DM (*p *< 0.05) and T2DM patients compared with the control group (*p *< 0.05), while no significant difference was observed between the pre‐T2DM subjects and the T2DM group (*p *> 0.05). The expression of miR‐126 was not statistically significant between the three groups (*p* > 0.05) (Figure 1).

### 3.4 Correlation between the expression of miR‐21, miR‐126, and the clinical and biochemical variables

Pearson correlation analysis showed that the miR‐21 expression was positively correlated with HbA1c (*r* = 0.456), FBS (*r* = 0.412), and TG (*r* = 0.278) (all *p *< 0.05). However, there was no significant correlation between miR‐21 and TC, SBP, DBP, HDL‐C, and LDL‐C (all *p *> 0.05) (Table 2). There was no significant correlation between miR‐126 expression level and clinical and biochemical factors (all *p *> 0.05). The assessment of the miR‐21 and miR‐126 expressions in different age and sex groups showed no significant difference (*p *> 0.05). Multiple linear regression analysis showed that the HbA1c was the main factor influencing the miR‐21 expression in plasma samples (*p *< 0.05). Independent variables of the equation and related parameter values are shown in Table 3.

**TABLE 2:** Correlation between the expression of miR‐21, miR‐126, and the clinical and biochemical characteristics in groups

Indicators	HbA1C	FBS	TC	TG	LDL	HDL	DBP	SBP
miR−21								
*r*	0.456*	0.412* ^,^ **	0.213	0.278*	0.182	−0.062	0.150	0.198^a^
*p*	<0.001	<0.001	0.060	0.013	0.108	0.585	0.188	0.080
miR−126								
*r*	0.019	0.011**	0.126	0.083	0.130	−0.087	−0.092	−0.056^a^
*p*	0.796	0.266	0.922	0.654	0.791	0.981	0.446	0.641

Note

Data are presented as mean ±SD unless otherwise noted. Data were evaluated using 2^ΔΔCt^ of miR‐21 and miR‐126 and variables by Pearson correlation/^a^Spearman correlation test.

Abbreviations: DBP, diastolic blood pressure; FBS, fasting blood sugar; HbA1c, glycosylated hemoglobin; HDL‐C, high‐density lipoprotein cholesterol; LDL‐C, low‐density lipoprotein cholesterol; SBP, systolic blood pressure; TC, total cholesterol; TG, triglyceride.

* *p* < 0.05

** *p*‐value was obtained after variables were log transformed to normalize the distributions.

**TABLE 3:** Multivariate linear regression analysis of miR‐21, miR‐126‐related factors

Variable	Regression coefficient	S.E.M.	Beta	t	*p* value
Constant(miR−21)	−1.708	1.003	–	−1.703	0.093
HbA1c	0.507	0.244	0.350	2.073	0.041
FBS	0.107	0.132	0.146	0.810	0.421
TG	0.002	0.004	0.017	0.126	0.900

Abbreviations: B, standardized regression coefficient; FBS, fasting blood sugar; HbA1c, glycosylated hemoglobin; TG, triglyceride.

### 3.5 Diagnostic value of miR‐21 in pre‐T2DM and T2DM individuals

**FIGURE 2:**
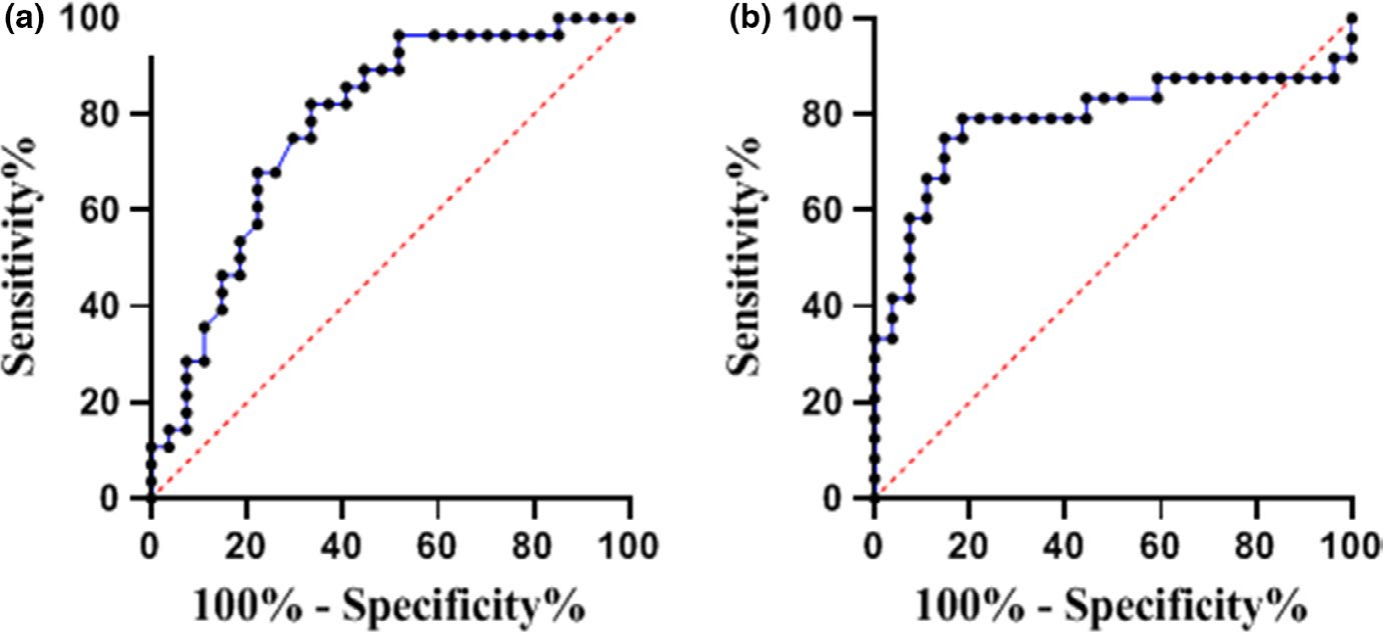
Receiver operating characteristic (ROC) curves for the capacity of the plasma miR‐21 to evaluate the diagnostic values for pre‐T2DM (a) and T2DM (b). ROC curves demonstrated an approximately equal level of diagnostic performance for both pre‐T2DM and T2DM groups with the healthy group with AUC of 0.77 (95% CI 0.65–0.90; *p* = 0.0004) and AUC of 0.78 (95% CI 0.0.64–0.92; *p* = 0.0042), respectively

ROC curve analysis was applied to measure the diagnostic value of circulating miR‐21 as a potential biomarker for pre‐T2DM and T2DM states. The results showed a reliable diagnostic value of miR‐21 (healthy vs. pre‐T2DM) with 0.77 (95% CI 0.65–0.90; *p *= 0.0004), 82.14%, and 66.67% and (healthy and T2DM) with 0.78 (95% CI 0.64–0.92%; *p *= 0.0042), 79.17%, and 81.48% of AUC, sensitivity, and specificity, respectively (Figure 2a,b). Importantly, there was an approximately equal level of diagnostic performance for both pre‐T2DM and T2DM groups, miR‐21 exhibited a potential biomarker value in prediabetes state.

## 4 DISCUSSION

miRNAs, as significant mediators of intracellular communication, and regulators in many biological processes (He et al., 2007; Kim & Kim, 2007; Yoo & Greenwald, 2005) may be ideal biomarkers to prognosticate and identify predisposition individuals for T2DM before the clinical symptoms begin.

This study reports the expression values of miR‐21 and miR‐126 in plasma of pre‐T2DM and T2DM individuals with a positive family history of diabetes. Also, we investigated the association of miR‐21 and miR‐126 with FBS, HbA1c, DBP, SBP, and lipid profiles as risk factors of cardiometabolic.

We demonstrated that circulating miR‐21 increased in pre‐T2DM and T2DM subjects. The ROC analysis of the relative expression of miR‐21 exhibited reasonable sensitivity and specificity (82.14%, 66.67%, and 79.17%, 81.48%) in discriminating pre‐T2DM and T2DM patients from healthy, besides. These results could provide novel insights into molecular biomarkers in subjects with genetic susceptibility to T2DM. The remarkable stability of circulating miRs in various circumstances, such as the presence of ribonucleases, freezing/thawing cycles, and other extreme experimental settings, are notable. Furthermore, plasma specimens are kept at −80^∘^C for several months, and miRs are conserved well in tissue samples even after paraffin‐embedding and formalin‐fixation, without significant degradation (Glinge et al., 2017; Li et al., 2007; Weickmann & Glitz, 1982).

In T2DM patients, miR‐21 has been reported to be either decreased or increased based on the sample type. Elevated expression of miR‐21 has been reported in diabetic complications such as cardiomyopathy (Al‐Hayali et al., 2019), nephropathy (Kölling et al., 2017), and retinopathy (Gui et al., 2020). Also, miR‐21 could be an early predictor of reactive oxygen species (ROS)‐mediate damage in subjects with a high risk of T2DM that is positively associated with glycemic parameters and ROS production (Conway et al., 2006). In addition, Seyhan et al. (2016) reported an increase in miR‐21 levels in plasma specimens of T2DM patients, which is significantly associated with HbA1c, β‐cell function, and insulin resistance. Contrary to our results, several studies have shown significantly decreased expression of miR‐21 among diabetes individuals (La Sala et al., 2019; Olivieri et al., 2015). Wang et al. (2014) reported a significant decrease in miR‐21 of T2DM Iraqi patients living in Sweden. Interestingly, due to a considerable reduction of miR‐21 in Iraqis compared to Swedish T2DM individuals, an ethnicity‐specific expression of miR‐21 was suggested.

Although in our study, the relative gene expression of miR‐126 was not different between the three groups. The other studies have shown that the miR‐126 level declined in T2DM patients (Rezk et al., 2016; Zhang et al., 2015). The inconsistent outcomes of relative expression of miRs to prior studies might root in various factors. It has been shown in different studies that miRs expression is affected by genetic background and alters between populations that share the same environment (Meerson et al., 2019; Wang et al., 2014). The accuracy of miRs measurement platforms and the high sequence similarity of miRs among family members are other factors that can play a considerable role in this discrepancy. Further challenges that apply additional inconsistency are identifying the disease stages and specific tissue expression in which investigation of miRs expression has been placed. For example, the investigation on the serum, peripheral blood mononuclear cells (PBMCs), and whole blood in populations of T2DM individuals with the same ethnicity has shown varying results of miRs expression (He et al., 2017). Obesity and gender‐specific effects are other confounding factors for T2DM (Meerson et al., 2019; Pescador et al., 2013).

In the present study, we observed no significant correlation between miR‐21 with SBP and DBP in T2DM individuals compared to the control group; this is probably because miR‐21 levels did not reach a sufficient threshold to show diabetes complications. Consistent with this study, Thum et al. (2008) established that miR‐21 is upregulated in cardiac fibroblasts in heart failure. In other studies, miR‐21 levels were increased in hypertensive rats compared with control rats, moreover, the miR‐21 level has elevated in hypertensive patients compared with controls, and correlations of miR‐21 expression level with 24‐h DBP and the dipping status have been observed (Kontaraki et al., 2014). In contrast, Li et al. (2016) demonstrated that blood pressure alleviated after delivering exogenous miR‐21 in the SHR mode. Furthermore, miR‐21 has a protective role in ischemia/reperfusion through reduction of cardiomyocytes apoptosis by targeting the PDCD4 mRNA (Qin et al., 2012). Dai et al. (2018) demonstrated that miR‐21 by reduction of gene expression of gelsolin, result in the preservation of diabetes cardiomyopathy. Despite the high number of researches meant to recognize miRs associated with diabetes, only a few of them detected a subset of potentially promising miRNAs. Because of different etiologies, such as genetics, lifestyle, and, environmental agents, T2DM is propounding diagnostic and therapeutic challenges.

In conclusion, this study indicates that the plasma expression level of miR‐21 can be considered as a non‐invasive and fast tool for distinguishing pre‐T2DM and T2DM individuals from healthy individuals. Nevertheless, the current study places the groundwork for future works to study miR‐21 as a unique class of blood‐based biomarkers in T2DM individuals. This study had some limitations including, the sample size and the study population. More investigation need in the future for expression of many inflammatory factors, genes involved in metabolism, oxidative stress, and even miRs polymorphism.

## CONFLICT OF INTEREST

The authors do not have any conflict of interest including any financial, personal, or other relationships with other people or organizations.

## AUTHOR CONTRIBUTIONS

**Zakieh Yazdanpanah** contributed to perform the experiments, data curation, write the manuscript, and support the statistical analysis of data. **Nasrin Kazemipour** contributed to project administration, funding acquisition, supervision, and correct and revision of the manuscript and submission. **Seyed Mehdi Kalantar** contributed to the conception/design and data collection. **Mohammad Yahya Vahidi Mehrjardi** contributed to analysis and data interpretation, conception, and design of the study. All authors read and approved the final manuscript.
